# The Expression Pattern and Functional Analysis of Extracellular Vesicle Long Non-Coding RNAs from Uterine Fluid During Implantation in Pig

**DOI:** 10.3390/ani15020245

**Published:** 2025-01-16

**Authors:** Yijun Shang, Qiuping Zhang, Yue Ding, Yongzhong Wang, Shengchen Gu, Xupeng Zang, Zheng Xu, Sixiu Huang, Zicong Li, Zhenfang Wu, Ting Gu, Linjun Hong, Gengyuan Cai

**Affiliations:** 1 State Key Laboratory of Swine and Poultry Breeding Industry, National Engineering Research Center for Breeding Swine Industry, College of Animal Science, South China Agricultural University, Guangzhou 510642, China; 2 Guangdong Provincial Key Laboratory of Agro-Animal Genomics and Molecular Breeding, South China Agricultural University, Guangzhou 510642, China; 3 Yunfu Subcenter of Guangdong Laboratory for Lingnan Modern Agriculture, Yunfu 527300, China; 4 National Regional Gene Bank of Livestock and Poultry (Gene Bank of Guangdong Livestock and Poultry), Guangzhou 510642, China

**Keywords:** pig, embryo implantation, extracellular vesicles, LNC_026212

## Abstract

Long non-coding RNAs (lncRNAs) act as competing endogenous RNAs and play significant roles in porcine embryo development. Extracellular vesicles (EVs) in the uterine fluid (UF) can target and deliver maternal endometrial signalling molecules to embryonic trophoblast cells, exerting crucial regulatory effects during embryo implantation. However, the specific roles of lncRNAs carried by UF-EVs during the embryo implantation period have not been thoroughly reported in the literature. In the present study, high-throughput sequencing and biological tools were applied to analyse lncRNAs in UF-EVs on days 9, 12, and 15 of pregnancy to identify key regulatory lncRNAs in UF-EVs during the porcine embryonic implantation period and to explore their expression patterns and functional roles. A total of 30,203 lncRNAs were identified and 7879 differentially expressed lncRNAs were screened, and qRT-PCR was used to verify the sequencing data. Days 9–12 of pregnancy represent a critical stage of embryo implantation characterised by substantial morphological changes in porcine embryos. During this period, we identified a total of 4348 differentially expressed lncRNAs. Through screening and validation, we discovered that LNC_026212 was highly expressed on day 12 of pregnancy and can promote the proliferation and migration of porcine trophoblast cells (PTr cells). These novel findings contribute to our understanding of the impact of lncRNAs on porcine reproductive processes, offering new research directions to improve the success rate of embryo implantation in pigs.

## Simple Summary

We discovered that LNC_026212 was significantly overexpressed in porcine uterine fluid extracellular vesicles (UF-EVs) on day 12 of pregnancy (P12). Our validation studies confirmed that LNC_026212 can significantly influence the proliferation and migration of porcine trophoblast cells (PTr cells). Furthermore, we predict that LNC_026212 may exert a regulatory function during the embryo implantation period by targeting the gene RBP4.

## 1. Introduction

The embryo survival rate is one of the primary factors determining litter size in sows, impacting the economic efficiency of the swine industry. During the 114 days gestation period in pigs, most embryonic deaths occur at 12–18 days of pregnancy [1,2]. Studies have shown that the embryonic mortality rate during early pregnancy ranges from 20 to 30% [2], which results in a significant waste of embryonic resources and limits the reproductive performance of sows. Therefore, researching the mechanisms of high reproductive capacity in sows is essential for exploring ways of reducing embryonic mortality during the implantation period, thereby improving the productivity of pigs.

Successful implantation requires the synchronisation of endometrial receptivity and embryonic competence for implantation [3]. It is important to note that the porcine placenta is classified as epithelial chorioallantoic placenta and the embryo does not invade the endometrium during implantation [4]. Before implantation, the embryo remains free-floating in the uterine fluid for an extended period. The maternal–fetal communication is dependent on the presence of the relevant factors in the uterine fluid, which can be described as the transport system for maternal–fetal communication and material exchange, mediating the transfer of various substances [5].

Extracellular vesicles (EVs) are a collective term for membrane-bound structures released by cells into the extracellular space and carry DNA, RNA, metabolites, cell surface proteins, lipids, and other substances [6,7]. The transported materials vary depending on the origin of the cells, the size of the EVs, and the recipient cells [8]. As mediators of intercellular signalling and material transport, EVs are widely distributed in biological fluids such as blood, breast milk, follicular fluid, and uterine fluid [8]. Previous research by our group has shown that extracellular vesicles in uterine fluid can selectively deliver maternal endometrial signalling molecules to embryonic trophoblast cells, playing a crucial regulatory role during embryo implantation [9,10]. Therefore, it is essential to further explore the regulatory mechanisms of the substances carried by uterine fluid extracellular vesicles on embryo implantation.

Long non-coding RNA (lncRNA) is one of the substances transported by EVs [11]. As nucleotide transcripts longer than 200 nt, lncRNAs are involved in various biological processes and diseases, such as muscle development [12], epigenetic regulation [13], and sex determination and differentiation [14]. They are essential for normal biological processes and for the onset and development of various complex diseases. In terms of embryo implantation, lncRNAs also play important roles. It has been shown that the downregulation of lncRNA H19 can increase miRNA let-7 activity, which can post-transcriptionally inhibit its downstream target, human integrin β3 (ITGB3), and thus weaken the adhesive and invasive ability of extrachorionic trophoblastic cells [15]. Transcriptome sequencing identified several differentially expressed lncRNAs that may play important roles in the implantation process of pig embryos [16]. Research has shown that lncRNA DANCR0 promotes trophoblast cell migration and invasion by downregulating miR-214-3p and activating the PI3K/AKT pathway [17].

Therefore, in this study, uterine fluid and uterine tissue were collected from Yorkshire sows at three stages: Days 9, 12, and 15 of pregnancy. Transcriptome sequencing was conducted to identify differentially expressed lncRNAs and investigate the mechanisms by which the lncRNAs affect embryo implantation. This experiment provides new information for further research on the regulatory mechanisms of lncRNAs in UF-EVs during embryo implantation. It contributes to understanding the impact of lncRNAs on porcine reproductive activities and offers new research insights to improve the success rate of embryo implantation.

## 2. Materials and Methods

### 2.1. Collection of Samples

This study was approved by the Ethics Committees of the Laboratory Animal Center of South China Agricultural University (permit number: SYXK-2022-0136).

Sows used for this experiment were described in Hu et al. [10]. In this study, healthy and disease-free Yorkshire sows of similar age and genetic background were selected. The sows were synchronised for estrus, and on the observed day of estrus, the sows were artificially inseminated, marking that day as P0. The day after insemination was considered P1 (day 1 of pregnancy), and so on. Uterus were obtained from sows slaughtered on days 9 (P9), 12 (P12), and 15 (P15) of pregnancy, with three sows per stage. Each uterine horn was cut approximately 70 cm from the tip of the uterine horn and washed with 200 mL PBS (Gibco, Grand Island, NY, USA) to collect uterine fluid. During uterine flushing, pregnancy was confirmed by the presence of normal spherical (P9) or filamentous (P12 and P15) embryos. The uterus was then opened to collect endometrial tissue. The collected samples were quickly frozen in liquid nitrogen and stored at −80 °C for further analysis.

### 2.2. EV Isolation

Methods for the isolation of extracellular vesicles were described in previous studies [9,10]. Separation of particles from UF was performed by ultracentrifugation. The supernatant was collected and centrifuged at 2000 rcf for 20 min at 4 °C to remove cells. It was then centrifuged at 10,000 rcf for 30 min at 4 °C to remove cell debris, large granular vesicles, and apoptotic bodies. The supernatant was then filtered through a 0.22 μm filter to remove contaminants. The filtered liquid was ultracentrifuged at 120,000 rcf for 2 h at 4 °C to collect the pellet. Finally, the pellet was resuspended in 100 μL PBS (Gibco, Grand Island, NY, USA) and stored at −80 °C.

### 2.3. Transmission Electron Microscope Assay

The resuspension of UF-EVs stored at −80 °C was thawed on ice, followed by pipetting 10 μL drops into a carbon-coated copper grid, and allowed to stand at room temperature for 2 min. The sample was then stained with uranyl acetate solution and allowed to air dry at room temperature. Finally, images were taken using transmission electron microscope (FEI Talos F200S).

### 2.4. Nanoparticle Tracking Analysis (NTA)

The ZetaView PMX120 (Particle Metrix, Munich, Germany) was used to track and count UF-EVs of different particle sizes suspended in phosphate-buffered saline (PBS). Nanoparticle tracking analysis (NTA) was employed to analyse the size of extracellular vesicle (EV) particles, and GraphPad Prism 8.0 was used for data visualisation.

### 2.5. Extraction of EV Proteins

For each 25 μL of EVs, add 10 μL of RIPA Lysis Buffer (CWBIO, Beijing, China). The mixture was incubated on ice for 20 min to allow for complete lysis of the EVs. Centrifuge the lysate at 12,000 rcf for 7 min at 4 °C. Carefully collect the supernatant and store it at −80 °C.

### 2.6. Western Blot

The EV proteins were denatured by heating and proteins were separated by SDS-PAGE gel (Servicebio, Wuhan, China) electrophoresis at a constant voltage of 120 V for 1 h. Subsequently, the proteins were transferred onto a PVDF membrane (Merck Millipore, Darmstadt, Germany) at a constant current of 0.2 A for 1.5 h. The membrane was blocked with 5% skimmed milk (Sangon, Shanghai, China) at room temperature on a shaker for 2 h and then washed with TBST (Servicebio, Wuhan, China). The membrane was incubated overnight at 4 °C with the following antibodies: anti-TSG101 (cat. no. 381538, ZENBIO, Chengdu, China, 1:1000), anti-HSP70 (cat. no. 10995-1-AP, Proteintech, Wuhan, China, 1:1000), and anti-Calnexin (cat. no. 10427-2-AP, Proteintech, Wuhan, China, 1:1000). The next day, the membrane was incubated with secondary antibody (HRP-conjugated Goat Anti-Rabbit IgG, BBI Life Sciences D110058, 1:10,000, Shanghai, China) at room temperature on a shaker for 1.5 h. The ECL Western blot kit (CWBIO, Beijing, China) was used to prepare the chemiluminescent solution, and the bands were visualised using the EC3 imaging system (UVP).

### 2.7. RNA Isolation and Library Construction

Total RNA was extracted from the samples using Trizol (Invitrogen, Carlsbad, CA, USA). The PrimeScript™ RT reagent kit with gDNA Eraser (Takara, Tokyo, Japan) was used for reverse transcription to obtain cDNA according to the supplier’s instructions, and the cDNA was stored at 4 °C for later use. RNA purity (OD260/280 and OD260/230 ratios) was measured using a NanoPhotometer^®^ spectrophotometer (IMPLEN, Westlake Village, CA, USA), and RNA integrity was determined using the RNA Nano 6000 Assay Kit of the Bioanalyzer 2100 system (Agilent Technologies, Santa Clara, CA, USA).

The first steps in the construction of the lncRNA library were the removal of rRNA. The Epicentre Ribo-Zero™ rRNA Removal Kit (Epicenter, Madison, Madison, WI, USA) was used to deplete ribosomal RNA (rRNA), and residual rRNA was removed using ethanol precipitation. The fragmented RNA and dNTPs were used for first-strand cDNA synthesis. During the synthesis of the second strand, dTTP was replaced by dUTP in the dNTPs. Subsequently, cDNA terminal repair, a tail addition, sequencing junction, and length screening were carried out. Finally, the second strand of cDNA containing U was degraded by USER enzyme for PCR amplification to obtain the library.

### 2.8. LncRNA Identification and Characterisation

We used HISAT2 (v2.0.4) to align the filtered reads to the reference genome. HTSeq software (v2.0.3) was used to perform quantitative analysis of known gene types for each sample of the species, based on expression statistics to obtain the expression levels of various gene types in the samples. StringTie (v1.3.1) was used for transcript assembly utilising the HISAT2 alignment results, allowing for the creation of the smallest possible set of transcripts and enabling quantitative analysis of transcripts. We then removed transcripts with exons ≤ 2 and transcripts with a length < 200 bp. Next, using Cuffcompare (v2.1.1), we filtered out transcripts that overlapped with annotated exon regions in the database. Transcripts from the database that overlapped with the exon regions of the newly assembled transcripts were included as annotated lncRNAs for subsequent analysis. We then used StringTie (v1.3.1) to calculate the Fragments Per Kilobase of exon model per Million mapped fragments (FPKM) values of lncRNAs and mRNAs in each sample, selecting transcripts with FPKM ≥ 0.5. Finally, we analysed the coding potential of the transcripts using three software tools: CNCI (v.2), Pfam-scan (v.1.3), and CPC (cpc-0.9-r2), ultimately identifying novel lncRNAs.

### 2.9. Differential Expression Analysis and Function Enrichment Analysis

We used edgeR for differential expression analysis. The threshold for differentially expressed (DE) genes was set at *p*-value < 0.05 and |log2FC| > 2. Based on gene expression data, principal component analysis (PCA) was used to analyse the similarity of the samples. Additionally, we calculated Pearson correlation coefficients to determine the differences within paired sample groups. Functional enrichment analysis of differentially expressed lncRNAs was performed using the R package Goseq for Gene Ontology (GO) analysis. This method accounts for gene length bias and GO terms with an adjusted *p*-value less than 0.05 were considered statistically significant difference. Additionally, KEGG pathway analysis was conducted using the KOBAS (2.0) software.

### 2.10. Target Gene Prediction

Cis-acting lncRNAs act on adjacent target genes. The co-location threshold is set to ±100 kb upstream and downstream of the lncRNA. Subsequent functional enrichment analysis of the mRNA genes within the co-location range will predict the primary functions of the lncRNA. The trans-acting role of lncRNAs was recognised by expression levels. Pearson correlation coefficients were used to analyse the correlation between lncRNAs and mRNAs across samples. mRNA genes with absolute correlation values greater than 0.95 were selected for functional enrichment analysis to predict the main functions of the lncRNAs. The R function cor.test was used to calculate the expression correlation between lncRNAs and coding genes. The trans-acting target genes of differentially expressed (DE) lncRNAs were predicted using BLAST (v2.2.28) software and RNAplex software (v2.7.0).

### 2.11. PPI Analysis

Differential expression of lncRNAs will be analysed through protein–protein interaction (PPI) network analysis using the STRING database https://cn.string-db.org/ (accessed on 1 June 2024), which includes data for the domestic pig (Sus scrofa) species. The database allows extraction of interaction data for the target gene set to construct the network. Visualisation and editing of the network will be performed using Cytoscape (v3.9.0). The MCODE plugin within Cytoscape will be utilised to identify key subnetworks.

### 2.12. Real-Time Quantitative PCR (RT-qPCR)

According to the instructions of SYBR Green Pro Taq HS Premix (Rox Plus) (ThermoFisher, Shanghai, China), the experiment was conducted with three technical replicates. The PCR reaction parameters were as follows: 40 cycles of PCR amplification, with each cycle consisting of denaturation at 95 °C for 10 s, annealing at 60 °C for 60 s, and extension at 72 °C for 45 s. The average threshold cycle (Ct) values for each target were determined using the sequence detection system software. Melting curve analysis was performed to confirm the specificity of amplification and the absence of primer dimers after each amplification run. For relative gene expression analysis, the Ct values of each gene were normalised using internal reference genes and control samples. The relative expression levels were calculated using the 2^−△△CT^ method. Variance homogeneity testing was conducted as part of the analysis. Primer sequence information is available in Supplementary Table S1.

### 2.13. Paraffin Section

Uterine tissue was collected and fixed in 4% paraformaldehyde for approximately 12 h. After fixation, the tissues were removed from the fixative solution for block trimming, and the trimmed uterine tissues were placed in a dehydrated tissue frame and rinsed under running water for approximately 24 h. After rinsing under running water, the uterine tissues were dehydrated in graded alcohol series. The dehydrated tissue was then placed in xylene I and II for clearing, with each step lasting approximately 15 min. After clearing, the tissue was sequentially immersed in 60 °C paraffin wax I and II for 1 h each. After the tissues were immersed in paraffin wax, the liquid embedding wax was poured into the embedding frame and then placed in the tissues for embedding. The embedded wax blocks were removed and trimmed, and the repaired wax blocks were cut with a slicer to produce slices approximately 4 μm thick. The sections were floated on 37 °C warm water to ensure proper flattening and then mounted onto anti-detachment slides. After drying, the slices were baked in an oven at 60 °C for about half an hour, and finally the baked sections were stored at 4 °C for preservation.

### 2.14. FISH Assay

Prepare paraffin sections of uteri from P9 and P12. Place the paraffin sections in BioDewax and cleaning solution (Servicebio, Wuhan, China) for 15 min, then dehydrate twice in anhydrous ethanol for 5 min, then immerse in PBS. Allow the sections to air dry naturally. Boil the sections in retrieval solution for 10 min, then let them cool naturally. Add Proteinase K (20 μg/mL) and incubate at 37 °C for 20 min for digestion. Rinse with distilled water, then wash in PBS three times for 5 min each. Add pre-hybridisation solution and incubate at 37 °C for 1 h. Remove the pre-hybridisation solution and add the probe-containing hybridisation buffer (Servicebio, Wuhan, China). Hybridise overnight at 37 °C in a constant temperature chamber. The next day, the hybridisation solution was washed off and washed with 2 × SSC (Servicebio, Wuhan, China) at 37 °C for 10 min; wash twice with 1 × SSC at 37 °C for 5 min each, followed by a wash with 0.5 × SSC at room temperature for 10 min. Finally, DAPI (Servicebio, Wuhan, China) staining solution was added dropwise and incubated in the dark for 8 min, rinse, and then anti-fluorescence quenching sealing tablets (Servicebio, Wuhan, China) were added. Observe and capture images using fluorescence microscope (Nikon, Tokyo, Japan).

### 2.15. Cell Culture and Transfection

Porcine trophoblast cells (PTr cells) isolated from porcine thread embryos on D12 of pregnancy. The PTr cells were cultured at 37 °C in a humidified incubator with 5% CO_2_, using DMEM/F12 (Gibco, Grand Island, NY, USA) supplemented with 0.1% insulin (YEASEN, Shanghai, China) and 10% fetal bovine serum (Gibco, Grand Island, NY, USA). When the cells reached 80% confluence in the culture dish, they were digested with 0.25% trypsin (Gibco, Grand Island, NY, USA) and passaged at a ratio of 1:3. Appropriate amounts of healthy PTr cells were seeded in a 24-well plate and cultured overnight in complete medium without antibiotics. When the cells reached 70% to 80% confluence, transfection was performed using the Lipofectamine 3000 Kit (Invitrogen, Carlsbad, CA, USA).

### 2.16. EdU Assay

Cell proliferation was assessed at 48 h using the BeyoClick™ EdU-555 Kit (Beyotime, Shanghai, China) according to the manufacturer’s instructions. A 2 × EdU working solution was prepared and added to the cell culture medium, and the cells were incubated for 16 h. After EdU labelling, the culture medium was removed and 500 μL of 4% paraformaldehyde (Shyuanye, Shanghai, China) was added to fix the cells at room temperature for 15 min. The fixative was then removed and each well was washed three times with PBS for 3–5 min each. The cells were permeabilised using immunostaining permeabilisation buffer containing Triton X-100 (Beyotime, Shanghai, China). Click Additive Solution was added and the cells were incubated for 30 min. The nuclei were stained with DAPI (Servicebio, Wuhan, China) and the slides were mounted for analysis.

### 2.17. Wound-Closure Assay

First, use a marker to draw intersecting horizontal and vertical lines on the back of the 24-well plate, approximately 1 cm apart. Seed the wells with cells and transfect the cells the following days. Use a pipette tip to make a scratch perpendicular to the horizontal lines on the back of the plate. Remove the cell culture medium and rinse three times with PBS (Gibco, Grand Island, NY, USA). Add serum-free medium and return the plate to the cell culture incubator. Photograph at 0 and 48 h.

### 2.18. Statistical Analysis

All data were tested for normality prior to statistical analysis. All experiments in this study were conducted with three biological replicates. Results analysis was performed using GraphPad Prism 8.0 or SPSS 25.0 software. Qualitative data were described as mean ± standard deviation (Mean ± SD). A Student’s *t*-test was used to analyse significant differences between different groups. * *p* < 0.05 indicated a significant difference.

## 3. Results

### 3.1. Separation and Identification of EVs

**Figure 1:**
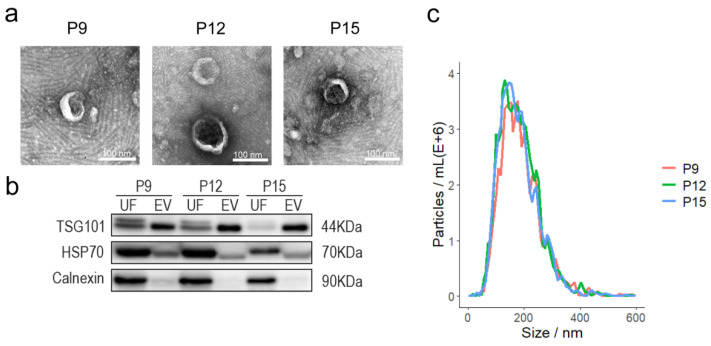
Characterisation of UF-EVs for identification. (**a**) Transmission electron microscopy images of UF-EVs on days 9, 12, and 15 of pregnancy; (**b**) Western blot analysis detected the marker proteins TSG101 and HSP70 in the EVs, with Calnexin used as negative controls. P9: day 9 of pregnancy, P12: day 12 of pregnancy, P15: day 15 of pregnancy; (**c**) nanoparticle tracking analysis (NTA) revealed the size of EVs.

Transmission electron microscopy (TEM) observed that the UF-EV samples exhibited membrane-bound vesicles with a central depression (Figure 1a), consistent with the morphological characteristics of EVs. Western blot results showed the presence of EV surface marker proteins TSG101 and HSP70 in both uterine fluid and EVs, while the endoplasmic reticulum marker protein Calnexin was only expressed in uterine fluid and not in EVs (Figure 1b). NTA showed that the particle size of EVs was mainly concentrated around 165 nm (Figure 1c). Taken together, these results indicated that the UF-EVs isolated in this experiment conform to the basic characteristics of EVs and were free of cellular contamination, which can be used for subsequent sequencing analysis of UF-EV-derived RNA.

### 3.2. High-Accuracy Sequencing Data

In this study, three biological replicates of each of the three stage samples were sequenced, for a total of nine Yorkshire sows’ UF-EVs used for RNA-seq. After filtering out raw reads containing adapters and low-quality reads, the remaining high-quality sequences still amounted to over 11 G of data (Supplementary Table S2). In general, the sequencing error rate at each base position should be less than 0.5%. In this sequencing, the sequencing error rate ranged between 0.02% and 0.28%, which was lower than 0.50%, indicating high sequencing accuracy. The GC content, which is the percentage of bases G and C out of the total base count, ranged from 36% to 56% in this study’s samples.

The sequencing error rate was mainly distributed in the first six bases of the initial sequence and at the end of the sequenced fragments, and the error rate gradually increased as the sequencing proceeded and the longer the length of the sequence, which was in line with the distribution of the sequencing error rate of RNA-seq technology (Supplementary Figure S1).

### 3.3. Identification and Characterisation of the lncRNAs and mRNAs

**Figure 2:**
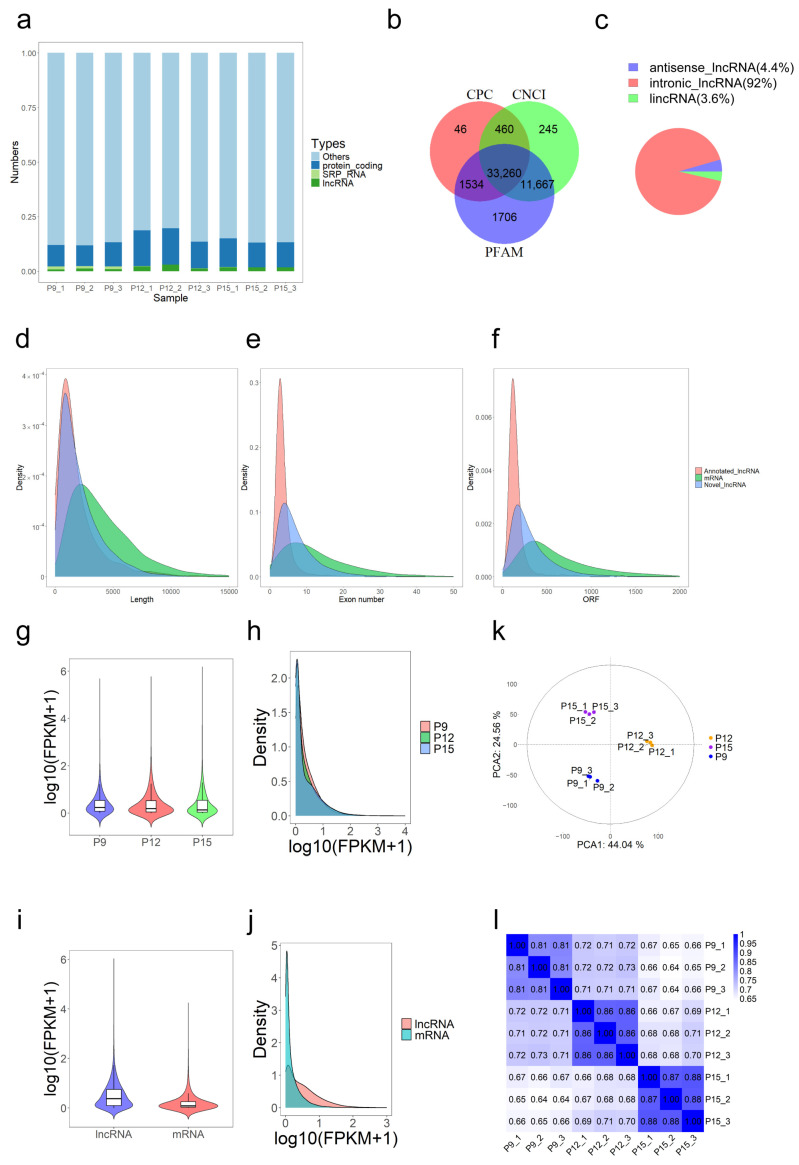
Identification and characterisation of the lncRNAs and mRNAs. (**a**) Distribution of reads across known gene types; (**b**) coding potential screening statistical chart. Three tools (CPC, CNCI, and PFAM) were used to analyse the coding potential of the lncRNAs. Overlapping circles indicated noncoding transcripts shared between software; (**c**) distribution plot of lncRNA types; (**d**–**f**) distribution of transcript length, number of exons, and ORF length in lncRNA and mRNA; (**g**) violin plot of lncRNA expression levels across different stages; (**h**) density distribution of FPKM values across different stages; (**i**) violin plot of lncRNA and mRNA expression levels; (**j**) density distribution plot of FPKM values for lncRNA and mRNA; (**k**) principal component analysis (PCA) plot; (**l**) Pearson correlation check among samples, where R2 represents the square of the Pearson correlation coefficient.

Among the aligned reads, known lncRNAs accounted for 0.80% to 3.00% of the total reads, rRNA accounted for less than 0.03%, protein-coding genes accounted for 9.50% to 16.70%, and miRNA accounted for less than 0.01% (Figure 2a). A total of 148,747 lncRNAs were identified through uterine fluid extracellular vesicle transcriptome sequencing, of which 33,260 were novel lncRNAs. Among the novel lncRNAs, intergenic lncRNAs accounted for 3.6%, antisense lncRNAs for 4.4%, and intronic lncRNAs for 92.0% (Figure 2b,c).

To further understand the differences between lncRNAs and mRNAs, we performed comparative structural analyses of novel lncRNAs with database-annotated known lncRNAs and mRNAs. lncRNAs were shorter than mRNAs in length as well as in exon number and open reading frames (Figure 2d–f). The average lengths of novel lncRNAs, annotated lncRNAs, and mRNAs were 2207.85, 1990.14, and 4259.41 nt, respectively. The characteristics of the novel lncRNAs identified in this study were consistent with the previously reported lncRNAs [18], which can be considered to be in line with the general features of known lncRNAs.

Violin plots and density distribution plots illustrated expression level differences across different stages (Figure 2g,h). Additionally, another set of violin plots and density distribution graphs showed the overall expression level differences between lncRNA and mRNA (Figure 2i,j). Among the three stage groups, the P12 group showed higher median expression levels, with lncRNAs exhibiting higher median expression levels compared to mRNAs.

Principal component analysis of lncRNA expression levels (Figure 2k). Samples were categorised into three classes, with distinct expression patterns of lncRNAs distinguishing samples within the same stage. The correlation analysis of expression patterns among the nine samples showed that the R^2^ values for each sample exceed 0.6 (Figure 2l).

### 3.4. Differentially Expressed LncRNAs

**Figure 3:**
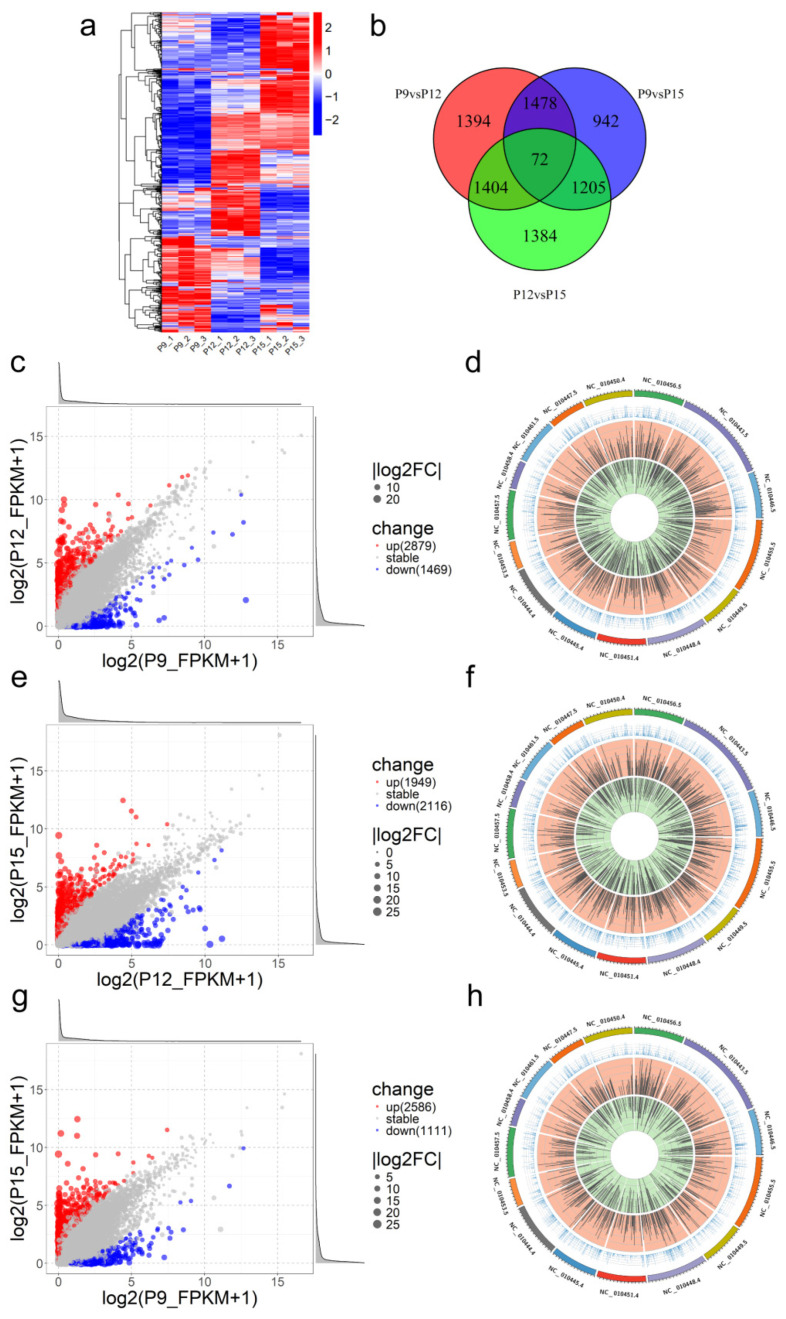
Differentially expressed LncRNAs. (**a**) The DE lncRNA clustering heatmap displayed an overall FPKM hierarchical clustering plot, clustered based on log10 (FPKM+1) values, where red indicated high expression genes and blue indicated low expression genes; (**b**) the Venn diagram of DE lncRNAs between groups; (**c**) the scatter plot of DE lncRNAs between P9 and P12; (**d**) the chromosome distribution plot of DE lncRNAs between P9 and P12. The outermost circle represented chromosomes (15 in total). The second circle represented the average FPKM values of the respective chromosomes in the comparison group. The third circle indicated the distribution of significantly upregulated transcripts on the chromosomes and the fourth circle showed the distribution of significantly downregulated transcripts on the chromosomes; (**e**,**f**) the scatter plot and chromosome distribution plot of DE lncRNAs between P12 and P15; (**g**,**h**) the scatter plot and chromosome distribution plot of DE lncRNAs between P9 and P15.

The DE lncRNA clustering heatmap demonstrated distinct enrichment clusters for DE lncRNAs across different stages (Figure 3a). There were a total of 72 lncRNAs that were differentially expressed across all three comparisons (Figure 3b).

In the comparison of P9 vs. P12, there were 4348 DE lncRNAs, with 2879 upregulated and 1469 downregulated (Figure 3c,d). For P12 vs. P15, there were 4065 DE lncRNAs, with 1949 upregulated and 2116 downregulated (Figure 3e,f). In the comparison of P9 vs. P15, there were 3697 DE lncRNAs, with 2586 upregulated and 1111 downregulated (Figure 3g,h). In the three comparisons, the distribution of differentially expressed lncRNAs on chromosomes is shown in Figure 3d,f,h.

### 3.5. Functional Enrichment Analysis of Differentially Expressed lncRNAs

**Figure 4:**
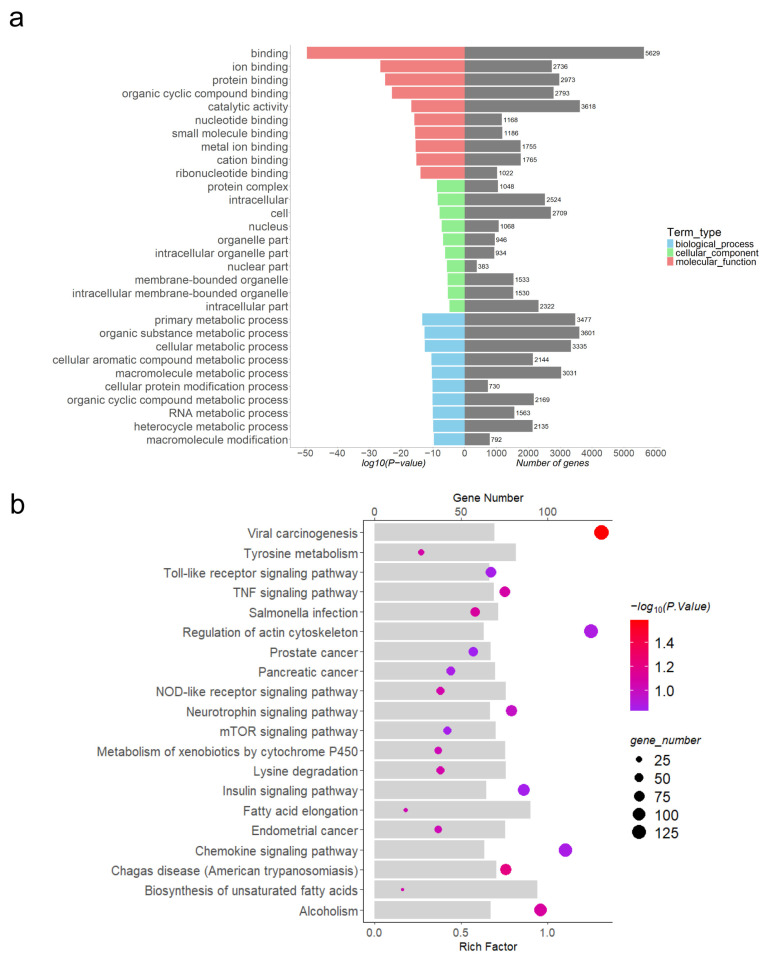
Functional enrichment analysis of cis-target genes for DE lncRNAs between P9 and P12. (**a**) GO analysis of cis-target genes of DE lncRNAs between P9 and P12; (**b**) KEGG analysis of cis-target genes of DE lncRNAs between P9 and P12.

**Figure 5:**
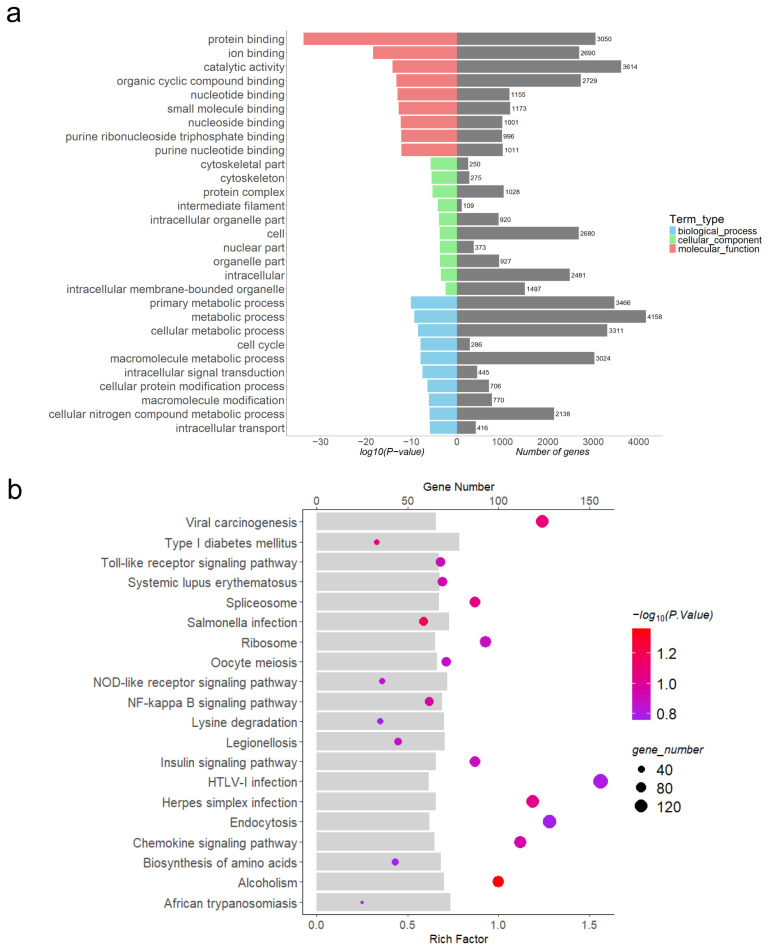
Functional enrichment analysis of cis-target genes for DE lncRNAs between P12 and P15. (**a**) GO analysis of cis-target genes of DE lncRNAs between P12 and P15; (**b**) KEGG analysis of cis-target genes of DE lncRNAs between P12 and P15.

**Figure 6:**
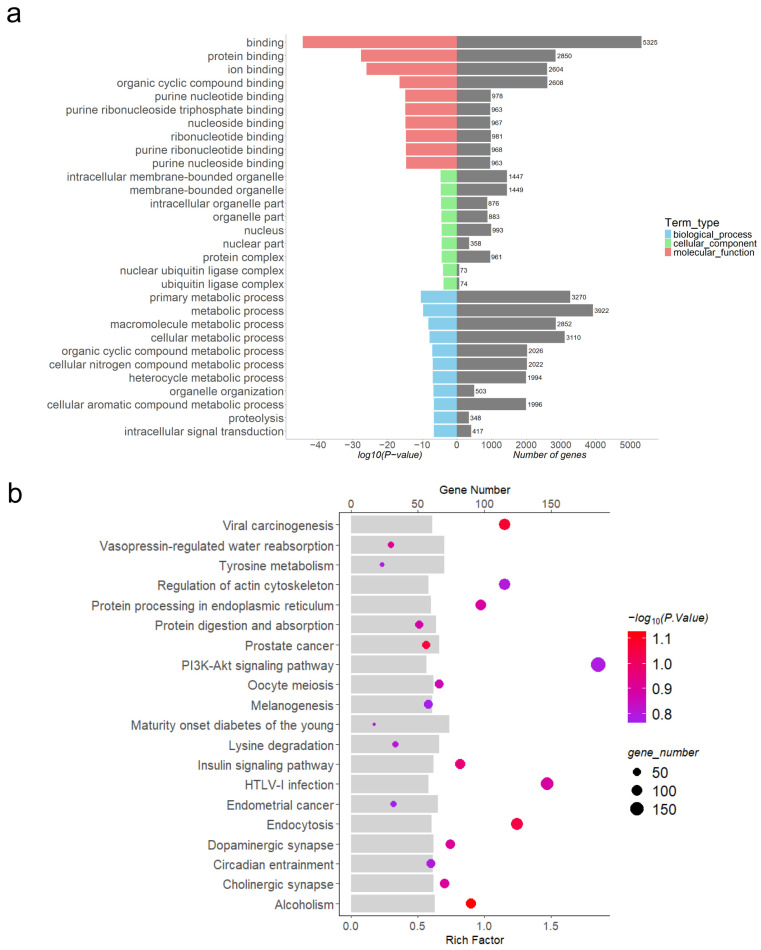
Functional enrichment analysis of cis-target genes for DE lncRNAs between P9 and P15. (**a**) GO analysis of cis-target genes of DE lncRNAs between P9 and P15; (**b**) KEGG analysis of cis-target genes of DE lncRNAs between P9 and P15.

Through software prediction, DE lncRNAs were found to cis-target 13,470 mRNAs and trans-target 13,610 mRNAs. To further understand the regulatory role of DE lncRNAs in porcine embryo implantation, GO functional analysis (Figure 4a, Figure 5a and Figure 6a, Supplementary Table S3) and KEGG pathway analysis (Figure 4b, Figure 5b and Figure 6b, Supplementary Table S3) were performed on DE lncRNA cis-target genes at each of the three stages. The pathways mainly enriched were related to embryo implantation, such as the TNF signalling pathway, mTOR signalling pathway, NF-kB signalling pathway, and chemokine signalling pathway.

### 3.6. PPI Network Analysis of Differentially Expressed lncRNA Target Genes

**Figure 7:**
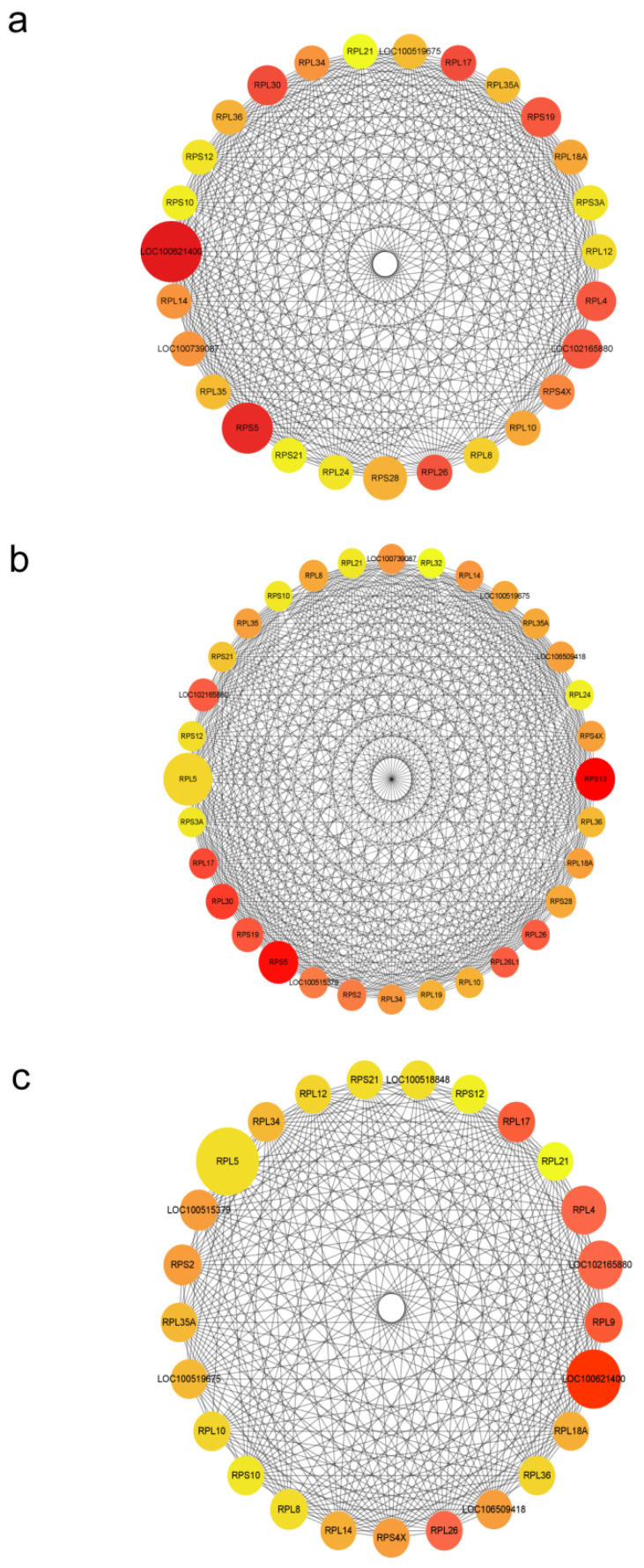
DE lncRNA cis-target gene PPI network. (**a**) PPI network of DE lncRNA cis-target genes between P9 and P12; (**b**) PPI network of DE lncRNA cis-target genes between P12 and P15; (**c**) PPI network of DE lncRNA cis-target genes between P9 and P15. Bigger betweenness values mean bigger nodes, and bigger degree values mean redder nodes.

The PPI network of DE lncRNA cis-target genes was constructed using Cytoscape software. The MCODE and cytoHubba plugins were employed to identify the highest-scoring PPI sub-networks within the DE lncRNAs, based on MCODE scores (Figure 7).

### 3.7. Screening of LNC_026212

**Figure 8:**
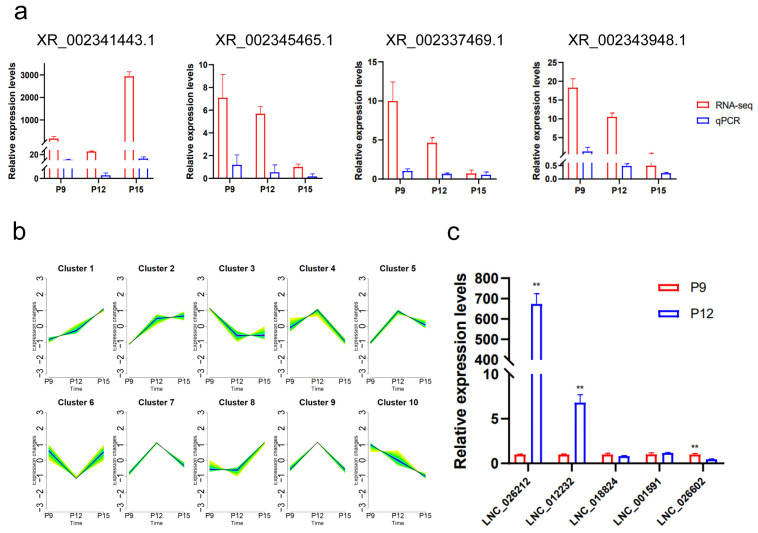
Screening of LNC_026212. (**a**) Validation of the expression of lncRNAs using quantitative real-time PCR (qRT-PCR); (**b**) temporal trend clustering plot; (**c**) the expression of candidate lncRNAs in the endometrial epithelium on P9 and P12. ** *p* < 0.01.

To verify the accuracy of the sequencing results, four differentially expressed lncRNAs were randomly selected to validate the sequencing results by qRT-PCR assay (Figure 8a). The validation results showed that the RT-qPCR results were consistent with the small RNA sequencing results, proving that the sequencing results were accurate and credible.

Based on the expression profiles of lncRNAs in UF-EVs across three different stages of pregnancy, the mfuzz software was utilised to cluster all Novel lncRNAs into 10 soft clusters based on their expression patterns (Figure 8b). It was hypothesised that lncRNAs playing crucial regulatory roles in embryonic implantation would exhibit significant upregulation from P9 to P12, with no significant expression differences between P12 and P15 stages. Based on this temporal expression pattern, Cluster 2, showing a temporal change trend during this period, was selected for further research and analysis.

Filtered differentially expressed lncRNAs from the enriched lncRNAs within the cluster 2 model. Then, filters were applied based on transcript expression levels with q < 0.05 and log2 fold change ≥ 1 between P12 and P9. Finally, these lncRNAs were sorted by descending order of FPKM values at P12, resulting in a total of five candidate lncRNAs (Supplementary Table S4). Studies have indicated that UF-EVs predominantly originate from the endometrial epithelium [10]. The relative expression levels of five candidate lncRNAs were validated by qPCR in the endometrial epithelium on P9 and P12 (Figure 8c). Among the candidate lncRNAs, LNC_026212 showed the highest relative expression in the endometrial epithelium on P12. Based on the combined results of transcript expression level estimation from sequencing data and qPCR experiments, LNC_026212 was selected for further investigation.

### 3.8. LNC_026212 Was Primarily Distributed in the Endometrial Luminal Epithelium and Glandular Epithelium

**Figure 9:**
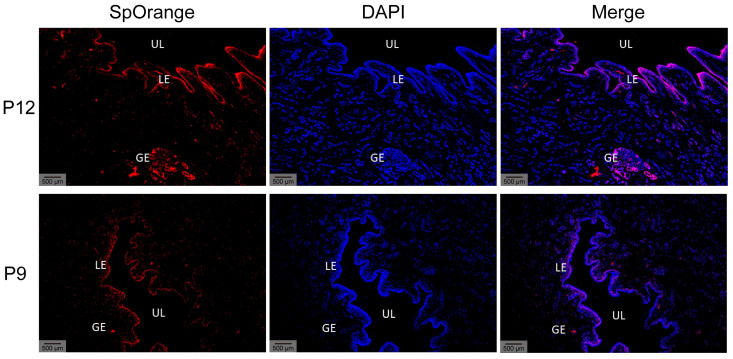
Distribution of LNC_026212 in the uterus on days 9 and 12 of pig pregnancy. Scale bar = 500 µm. LE: luminal epithelium; GE: glandular epithelium; UL: uterine lumen.

Fluorescence in situ hybridisation scans of uterine sections on days 9 and 12 of pregnancy showed that LNC_026212 was primarily distributed in the endometrial luminal epithelium and glandular epithelium, indicating that UF-EVs LNC_026212 may be secreted by endometrial epithelial cells (Figure 9).

### 3.9. Overexpression of LNC_026212 Promoted PTr Cell Proliferation and Migration

**Figure 10:**
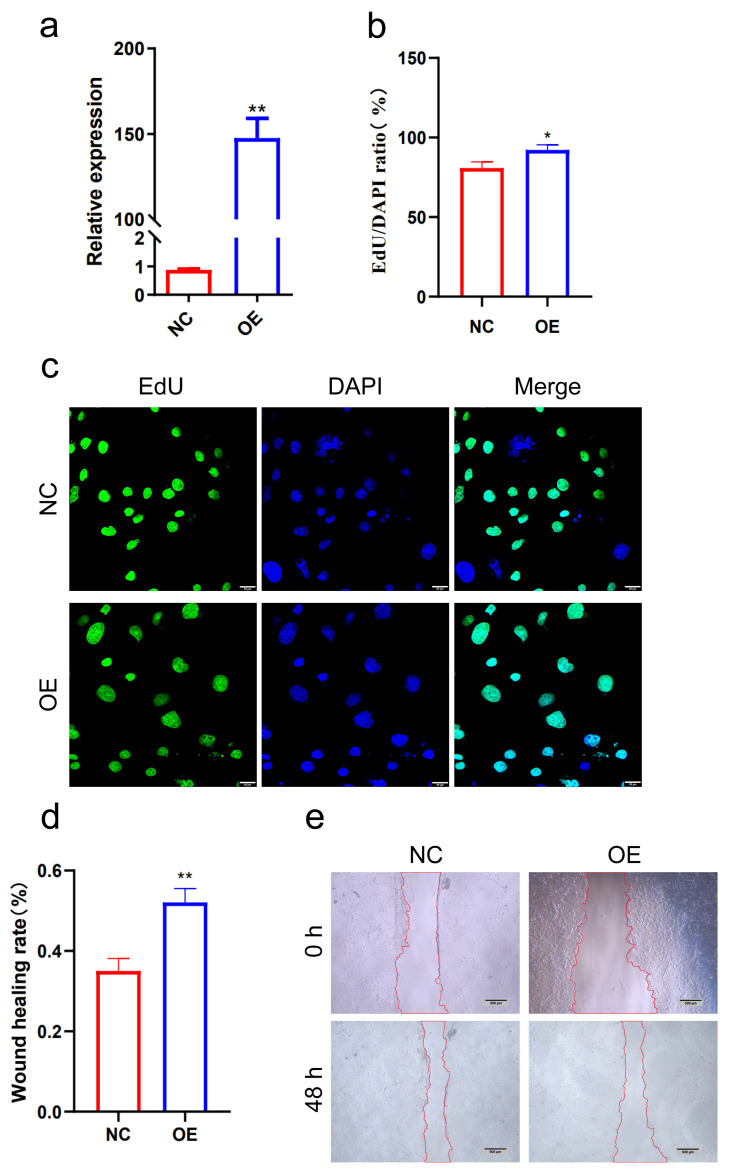
Overexpression of LNC_026212-promoted PTr cell proliferation and migration. (**a**) Expression efficiency of PTr overexpressing LNC_026212 detected by qPCR; (**b**,**c**) EdU assay showed altered cell proliferation after overexpression of LNC_026212. Scale bar = 25 µm; (**d**,**e**) the wound-closure assay to assess cell migration. Images were taken at 0 h and 48 h after cell damage. Scale bar = 500 µm. * *p* < 0.05; ** *p* < 0.01.

To investigate the regulatory effects of LNC_026212 on the function of porcine trophectoderm (PTr) cells, EdU and wound-closure assays were conducted to assess the impact of LNC_026212 overexpression vector, pCDNA3.1(+) empty vector, siRNA, and siNC on PTr cell proliferation and migration. QPCR results showed that the expression level was significantly upregulated in the overexpression plasmid group compared to the empty vector group (*p* < 0.01) (Figure 10a). The EdU assay confirmed that the overexpression of LNC_026212 significantly increased the number of EdU-positive cells in PTr cells (*p* < 0.05) (Figure 10b,c). The wound-closure assay demonstrated that the upregulation of LNC_026212 significantly increased the wound-healing rate of PTr cells and promoted cell migration (*p* < 0.01) (Figure 10d,e).

### 3.10. Inhibition of LNC_026212 Inhibited Proliferation and Migration of PTr Cells

**Figure 11:**
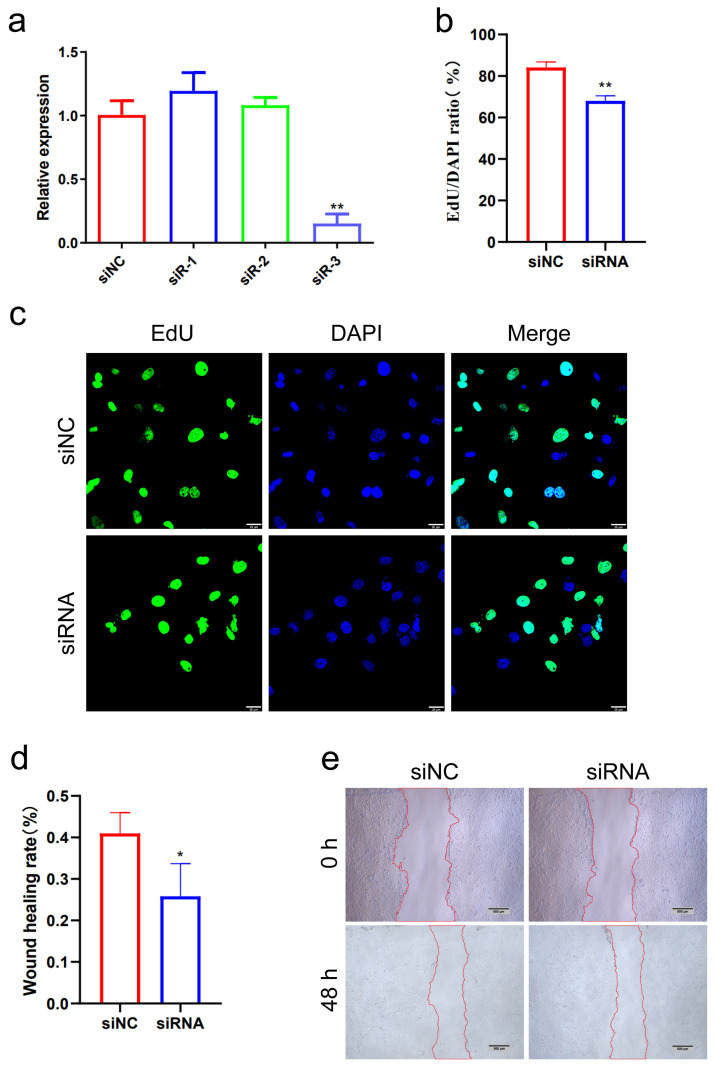
Inhibition of LNC_026212 inhibited PTr cell proliferation and migration. (**a**) qPCR was used to assess the inhibition efficiency of LNC_026212 in PTr cells under different treatments (siNC, siR-1, siR-2, siR-3); (**b**,**c**) EdU assay showed altered cell proliferation after inhibition of LNC_026212. Scale bar = 25 µm; (**d**,**e**) the wound-closure assay to assess cell migration. Images were taken at 0 h and 48 h after cell damage. Scale bar = 500 µm. * *p* < 0.05; ** *p* < 0.01.

The qPCR results showed that the expression of LNC_026212 was significantly downregulated in the siR-3 compared with the siNC (*p* < 0.01), Therefore, siR-3 was selected for the inhibition of LNC_026212 (Figure 11a). The EdU assay confirmed that the inhibition of LNC_026212 significantly reduced the number of EdU-positive cells in PTr cells (*p* < 0.05) (Figure 11b,c). The wound-closure assay demonstrated that the inhibition of LNC_026212 significantly inhibited the wound-healing rate of PTr cells and promoted cell migration (*p* < 0.01) (Figure 11d,e).

Combining the results of the EdU assay and the wound-closure assay, overexpression of LNC_026212 was shown to promote the proliferation and migration of PTr cells. Conversely, inhibition of LNC_026212 had an inhibitory effect on the proliferation and migration of PTr cells.

### 3.11. Prediction of LNC_026212 Cis-Regulated Target Genes

**Figure 12:**
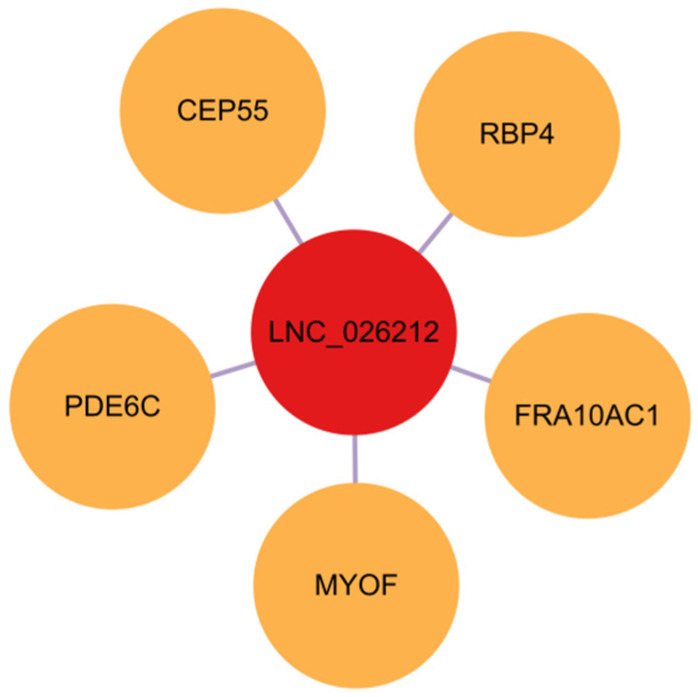
The cis-regulatory target gene network of LNC_026212.

In this study, we predicted five cis-regulatory target genes for LNC_026212: RBP4, FRA10AC1, MYOF, CEP55, and PDE6C (Figure 12). Among these, retinol-binding protein 4 (RBP4) exhibits a sequence similarity of 99.89% with LNC_026212 (Supplementary Table S5). Studies have shown that RBP4 influences the implantation process of pig embryos by regulating proliferation, migration, and apoptosis of endometrial epithelial cells and embryonic stem cells [3]. Therefore, it was speculated that LNC_026212 exerted regulatory functions during the porcine embryo implantation process through RBP4.

## 4. Discussion

Embryo implantation involves complex processes accompanied by the expression of various factors including growth factors, adhesion-promoting molecules, and cytokines, regulated by various lipids and hormones, such as progesterone, estradiol, and prostaglandins [19]. However, recent studies have reported that extracellular vesicles (EVs) play a crucial role in intercellular communication [20]. Some studies have found that EVs are associated with early pregnancy in pigs [21,22], and that EVs derived from embryos, placenta, and endometrium can mediate maternal–fetal communication, playing an essential role in early pregnancy [23,24]. Therefore, the aim of this study was to explore the expression profile of lncRNAs in uterine fluid (UF) to understand the role of lncRNAs in embryo implantation.

Embryo implantation typically involves interactions and signal transmissions between the embryo and the uterine endometrium. First, porcine embryos undergo rapid morphological changes from ovoid to filamentous structures between days 9 and 12 of pregnancy. At 12–13 days of gestation, blastocysts acquire the ability to implant, and the hormonal changes and immune responses in the uterus reach critical points [25]. During the embryo implantation period, day 12 of pregnancy is a critical time point that significantly affects embryo survival. In Chinese Taihu pigs, embryos exhibit a good implantation status on P12, which reduces the overall embryo mortality rate [26]. Research indicates that embryo–maternal recognition in pigs during early pregnancy is a complex and dynamic regulatory process that primarily occurs between days 11 and 13 of pregnancy. During this period, various molecular and cellular signals mediate interactions between the embryo and the mother to ensure successful implantation and the continuation of pregnancy [27]. Therefore, in this study, the focus was more on the sequencing data from P9 and P12 among the three stages.

Differential expression analysis between P9 and P12 identified 4348 significantly differentially expressed lncRNAs (*p* < 0.05), including 2879 upregulated and 1469 downregulated ones, indicating that numerous lncRNAs play regulatory roles during the implantation process from P9 to P12. We aimed to predict the cis_target genes for these DE lncRNAs and subsequently performed Gene Ontology (GO) functional analysis on the predicted mRNA targets. Among the top 10 enriched GO terms, molecular function (MF) mainly included various substance bindings such as protein binding, organic cyclic compound-binding, and metal ion-binding. Cellular component (CC) terms included protein complexes, intracellular, and nucleus. Biological process (BP) terms included primary metabolic processes, organic cyclic compound metabolic processes, RNA metabolic processes, and cellular protein modification processes. The GO results indicated extensive substance exchange during the implantation process from P9 to P12 of pregnancy. Performing KEGG pathway analysis on the target genes of DE lncRNAs can further infer the regulatory pathways through which lncRNAs exert their functions. Enriched pathways were closely related to embryo implantation, such as the tumour necrosis factor (TNF) signalling pathway, mTOR signalling pathway, and Nod-like receptor (NLR) signalling pathway. TNF can bind to TNFR1 on the cell membrane, activating TAK1 and MKK signalling pathways, which in turn activate MAPK and Wnt signalling pathways within cells. Ultimately, these signals are transmitted to downstream response molecules to regulate biological processes such as differentiation, proliferation, apoptosis, and necrosis. TNF-α served as a crucial messenger mediating interactions between the trophectoderm and uterine epithelial cells [28]. Leukaemia inhibitory factor (LIF) plays a regulatory role in cell proliferation and apoptosis in different cells, predominantly via induction of the MAPK signalling pathway during maternal acceptance of embryo implantation and placental formation [29,30]. Studies have shown that specific Wnt signalling pathway members essential for uterine events such as decidualisation, formation of uterine glands, and periodic changes in the endometrium controlled by reproductive hormones [31].

By P15 of gestation, embryos further elongate to filamentous embryos measuring 800 to 1000 mm in length [32]. Thereafter, the rate of embryonic elongation slows down, entering a relatively slower developmental stage. By around P18, the embryos make contact with the uterine epithelial lining and the placenta begins to be established [33]. Therefore, we performed GO and KEGG analysis of 4065 DE lncRNAs from P12 vs. P15. The top 10 enriched GO terms in the molecular function (MF) category primarily included binding activities such as protein binding, ion binding, and organic cyclic compound-binding. Additionally, catalytic activity was prominent, indicating the gene products’ capability to catalyse chemical reactions. In the cellular component (CC) category, terms mainly encompassed cytoskeleton, protein complex, and intracellular organelle parts. In the biological process (BP) category, the terms were mainly associated with organic substance metabolic processes, primary metabolic processes, cellular metabolic processes, and intracellular signal transduction processes. Among the top 20 enriched KEGG pathways, several pathways were closely related to embryonic development including biosynthesis of amino acids, NF-kB signalling pathway, chemokine signalling pathway, and TLR signalling pathway. Studies have shown that the NF-kB signalling pathway is essential for the early mesoderm differentiation of mouse embryonic stem cells [34]. Researchers have observed elevated levels of nitric oxide (NO) in the embryos of diabetic animals. NO is produced by nitric oxide synthases (NOS), including NOS1, NOS2, and NOS3. Studies have shown that the expression of the NOS2 gene is regulated by the NF-kB signalling pathway. The use of quercetin-3-glucoside reduces the incidence of neural tube defects in diabetic mouse embryos, suggesting that quercetin-3-glucoside may regulate the expression of NOS2 through the modulation of the NF-kB transcriptional regulatory system [35]. PGN is a specific ligand for TLR-2. Research has found that PGN can significantly increase the production of IL-1β, IL-6, and IL-23 by dendritic cells (DCs) and enhance their ability to promote the generation of IL-17^+^ uveitis-producing T cells [36]. Additionally, interleukins (ILs) play a crucial role in maternal–fetal recognition [37]. Chemokines are a group of small cytokines expressed on both sides of the maternal–fetal interface. Previous studies in humans have shown that chemokine ligands such as CXCL8 and CXCL1 induce trophoblast migration and invasion into the decidua. Therefore, the downregulation of these cytokines in the placenta may contribute to fetal loss and fetal growth restriction [38].

We also performed differential and functional enrichment analyses of lncRNAs in globular embryos on P9 and filamentous embryos on P15, identifying a total of 3697 DE lncRNAs. Among the top 10 enriched GO terms, biological process (BP) mainly included organic substance metabolic processes, cellular metabolic processes, and macromolecule metabolic processes. Among the enriched KEGG pathways, several pathways were closely related to embryo implantation, such as regulation of actin cytoskeleton, PI3K-Akt signalling pathway, and insulin signalling pathway. The actin cytoskeleton is involved in most cell movements, including embryonic morphogenesis, angiogenesis, and tissue repair and regeneration [39]. The PI3K/Akt signalling pathway is a well-known mediator of growth and cell survival signals. AKT1 is widely expressed in the mouse placenta, including trophoblast cells and vascular endothelial cells. Mice with AKT1 gene defects show abnormalities in placental development, including reduced angiogenesis, malnutrition, absence of glycogen cells in spongiotrophoblasts, and reduced decidua basalis [40]. In addition, the PI3K/Akt pathway is associated with trophoblast cell differentiation and migration [41]. PI3K/Akt pathway is present and functional in mouse pre-implantation embryos [42]. Some studies have found that insulin and insulin-like growth factor-1 (IGF-1) promote glucose uptake during the blastocyst stage by activating the insulin-like growth factor-1 receptor (IGF-1R). This process relies on the PI3K/Akt signalling pathway, and reduced activity of the IGF-1R or the PI3K/Akt signalling pathway can lead to impaired glucose uptake, potentially causing apoptosis of embryonic cells [42]. It was evident that the placental insulin, mTOR, and STAT3 signalling pathways are positive regulators of placental amino acid transporters. Activation of the placental insulin/IGF-I/mTOR and leptin pathways in obese mice stimulates placental amino acid transport and promotes fetal growth [43].

Total novel lncRNAs were clustered into 10 soft clusters based on expression patterns using mfuzz software (http://mfuzz.sysbiolab.eu/). We focused on the lncRNAs that were significantly upregulated in expression from P9 to P12, and at the same time had no significant expression difference from P12 to P15. Based on this expression pattern, we selected the expression trend cluster 2 for further investigation. Five lncRNAs were identified, and ultimately, LNC_026212, which had the highest expression of P12 among the differentially expressed lncRNAs of P9 and P12, was selected as a candidate lncRNA for embryo implantation for further validation analysis. In situ hybridisation experiments revealed that LNC_026212 was primarily distributed in the endometrial luminal epithelium and glandular epithelium, suggesting that UF-EVs LNC_026212 may be secreted by endometrial epithelial cells. EdU assay and wound-closure assay showed that the overexpression of LNC_026212 promoted proliferation and migration of PTr cells, whereas inhibition of LNC_026212 inhibited these processes. Both the qPCR and cell experiments together demonstrated that LNC_026212 plays an important regulatory role in embryo proliferation and migration during the implantation process.

Bioinformatics software predicted that the target gene of LNC_026212 is RBP4, as shown in Figure 12. In pigs, RBP4 is located on chromosome 14, and its cDNA is 937 bp long, encoding a mature protein of 176 amino acids [44]. RBP4 is a specific carrier protein that transports vitamin A from the liver to target tissues, facilitating the intracellular transport and metabolism of vitamin A. Previous studies have shown that gene expression in the retinoic acid signalling pathway is altered by decidualisation, indicating that RBP4 may be an important paracrine factor secreted by human decidualised endometrial stromal cells that activates the expression of the estradiol-metabolising enzyme HSD17B2 in the epithelium, leading to a reduction in local estradiol levels, highlighting the critical role of RBP4 in the process of embryo implantation [45]. The rapid morphological development phase of porcine blastocyst elongation is crucial for embryo survival, during which the expression level of RBP4 increases [44]. Similarly, the European roe deer undergoes pre-implantation embryo elongation. The team of van der Weijden found that RBP4 is involved in cell proliferation and tissue remodelling during this process. RBP4 expression in the endometrium of elongating embryos is significantly upregulated compared to the diapause period [46], consistent with the upregulation of RBP4 during porcine pregnancy reported by Wang’s team [47].

We identified the target gene RBP4 through bioinformatics analysis. Next, we plan to validate the regulatory relationship between LNC_026212 and RBP4. Subsequently, cellular functional assays will be conducted in PTr cells to determine whether RBP4 can influence their proliferation and wound-healing capacity. Finally, mouse uterine injection experiments will be performed to assess the effect of RBP4 on embryo implantation.

## 5. Conclusions

In this study, we performed genomic sequencing on UF-EVs collected on days 9, 12, and 15 of pregnancy to identify the expression profiles of mRNA and lncRNA. Among the identified lncRNAs, LNC_026212 was highly expressed on P12 of pregnancy and can promote PTr cell proliferation and migration.

## Data Availability

The raw sequence data reported in this paper were deposited in the Genome Sequence Archive [48] in the National Genomics Data Center [49], China National Center for Bioinformation/Beijing Institute of Genomics, Chinese Academy of Sciences (GSA: CRA018777) that are publicly accessible at https://ngdc.cncb.ac.cn/gsa (accessed on 4 September 2024).

## Supplementary Materials

The following supporting information can be downloaded at: https://www.mdpi.com/article/10.3390/ani15020245/s1, Figure S1: error rate; Figure S2: original Western blot for Figure 1b; Table S1: primer sequence for the Genes for Real-time RT-PCR; Table S2: summary of data output quality status; Table S3: GO and KEGG analysis of cis-target genes of DE lncRNAs; Table S4: list of candidate lncRNAs; Table S5: the top 5 alignment results of LNC_026212 with the pig genome; Table S6: FPKM values of DE lncRNAs in the uterine luminal fluid-EVs.
