# Supplementary material for: The Expression Pattern and Functional Analysis of Extracellular Vesicle Long Non-Coding RNAs from Uterine Fluid During Implantation in Pig
Source: Animals (Basel). 2025 Jan 16;15(2):245. doi: 10.3390/ani15020245 (PMC11758334; doi:10.3390/ani15020245)
Supplement: Supplementary file 1 [file animals-15-00245-s001.zip › Supplementary Figure S2 original western blot.pdf]

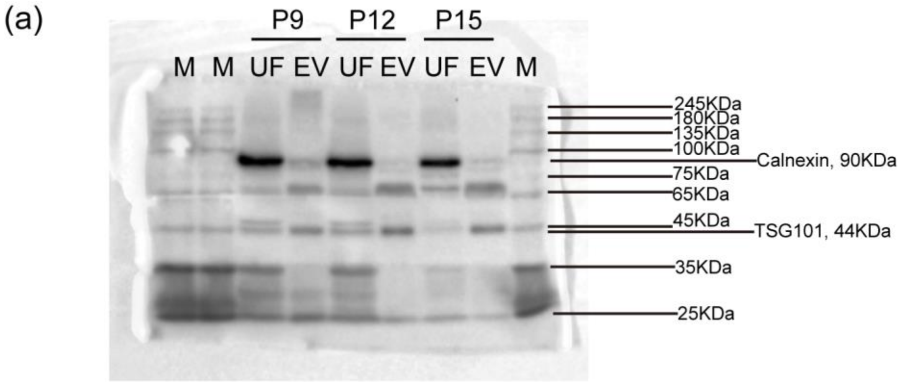

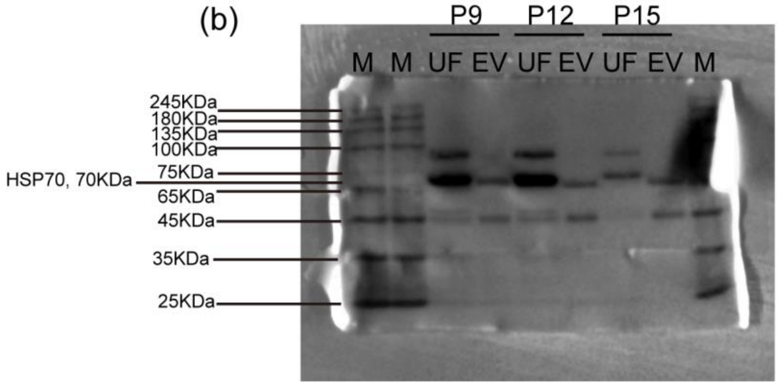

Figure S2. Original western blot figures for Figure 1b. (a) Calnexin, 90KDa, TSG101, 44KDa. (b) HSP70, 70KDa. UF: uterine fluid, EV: extracellular vesicle, P9: Day 9 of pregnancy, P12: Day 12 of pregnancy, P15: Day 15 of pregnancy, M: marker.
