# Supplementary figures and images for: The Expression Pattern and Functional Analysis of Extracellular Vesicle Long Non-Coding RNAs from Uterine Fluid During Implantation in Pig
Source: Animals (Basel). 2025 Jan 16;15(2):245. doi: 10.3390/ani15020245 (PMC11758334; doi:10.3390/ani15020245)

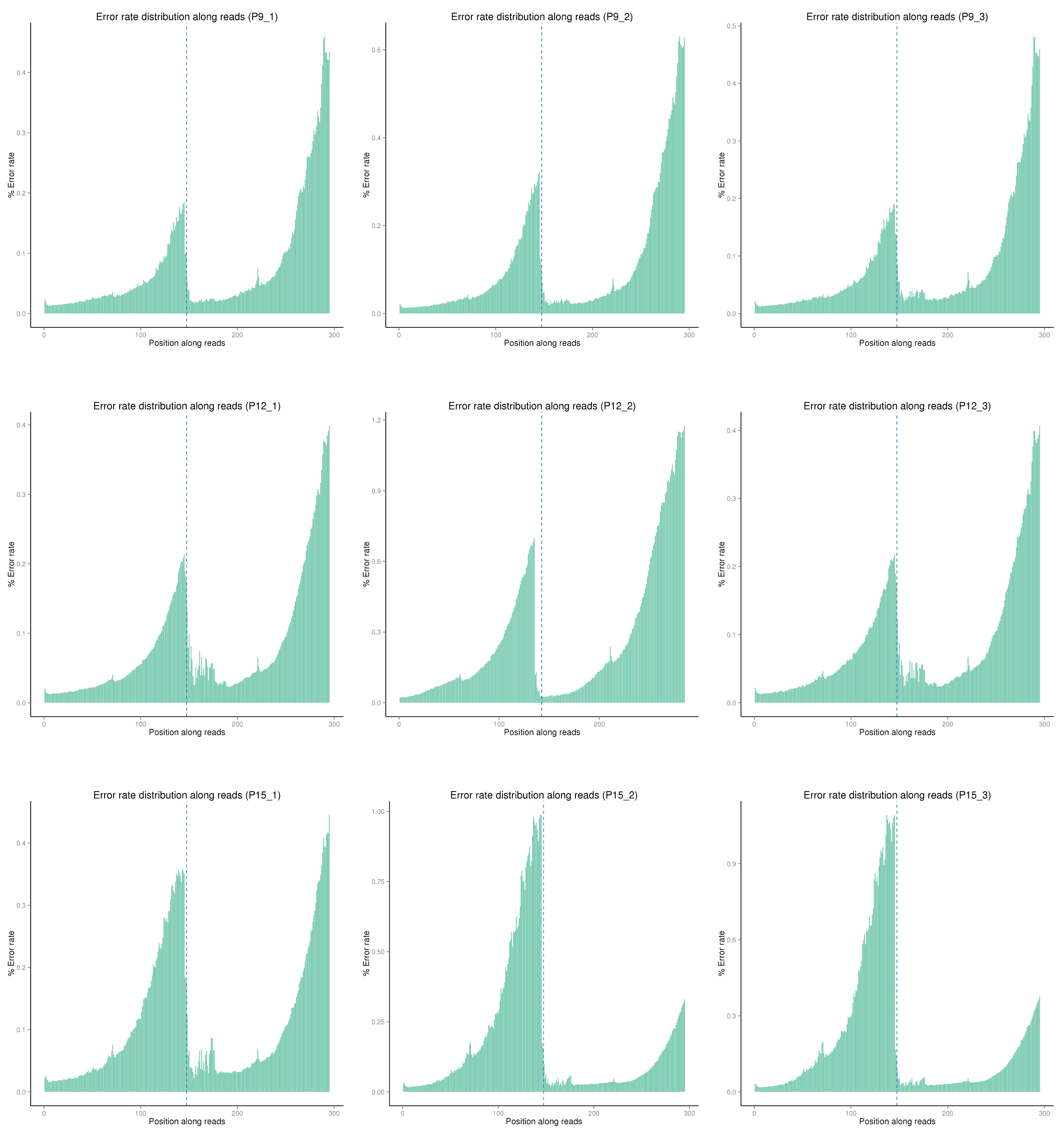

Supplement: Supplementary file 1 [file animals-15-00245-s001.zip › Supplementary Figure S1.tif]
